# “Cervical cancer screening: awareness is not enough”. Understanding barriers to screening among women in West Cameroon—a qualitative study using focus groups.

**DOI:** 10.1186/s12978-021-01186-9

**Published:** 2021-07-09

**Authors:** Alida Manoëla Datchoua Moukam, Muriel Samartha Embolo Owono, Bruno Kenfack, Pierre Vassilakos, Patrick Petignat, Jessica Sormani, Nicole C. Schmidt

**Affiliations:** 1 Department of Obstetrics and Gynecology and Maternal Heath, University of Dschang, Dschang District Hospital, Dschang, Cameroon; 2 Faculty of Medicine and Pharmaceutical Sciences, Department of Public Health and Epidemiology, University of Dschang, Dschang, Cameroon; 3 Gynecology Division, Department of Pediatrics, Obstetrics and Gynecology, University Hospitals of Geneva, Geneva, Switzerland; 4 Geneva Foundation for Medical Education and Research, Geneva, Switzerland; 5 Geneva School of Health Sciences, HESSO University of Applied Sciences and Arts Western Switzerland, Geneva, Switzerland; 6 Faculty of Social Science, Catholic University of Applied Science, Preysingstr. 95, 81667 Munich, Germany

**Keywords:** Cervical cancer, Prevention, Sub-Saharan Africa, Health literacy, Barriers

## Abstract

**Background:**

Cervical cancer is the second leading cause of cancer-related death among women in sub-Saharan countries, constituting a major public health concern. In Cameroon, cervical cancer ranks as the second most common type of cancer among women and the leading cause of cancer-related deaths, mainly due to the lack of prevention.

**Objectives:**

Our first and main objective was to understand the barriers affecting women’s decision-making process regarding participation in a cervical cancer screening program in the Dschang district (West Cameroon). Second, we aimed to explore the acceptability and perception of a single-visit approach (screen and treat).

**Methods:**

A qualitative study using focus groups (FGs) was conducted from February to March 2020. Female participants aged between 30 and 49 years and their male partners were invited to participate. Thematic analysis was used, and barriers were classified according to the three-delay model of Thaddeus and Maine.

**Results:**

In total, six FGs with 43 participants (31 women and 12 men) were conducted. The most important barriers were lack of health literacy, low accessibility of the program (in respect to cost and distance), and disrespectful treatment by healthcare workers.

**Conclusions:**

Our study identified three needs: (1) enhancing health literacy; (2) improving the delivery of cervical cancer screening in rural areas; and (3) providing training for healthcare providers and community healthcare workers to improve patient-provider-communication.

*Trial registration* Ethical Cantonal Board of Geneva, Switzerland (CCER, N°2017-0110 and CER-amendment n°3) and Cameroonian National Ethics Committee for Human Health Research (N°2018/07/1083/CE/CNERSH/SP). NCT: 03757299

## Plain Language Summary

Cervical cancer is the second leading cause of cancer-related death among women in sub-Saharan countries, constituting a major public health concern. In Cameroon, cervical cancer ranks as the second most common type of cancer among women and is the leading cause of cancer-related deaths, mainly due to the lack of prevention measures, such as cervical cancer screening.

The main aim of the current study was to understand barriers that affect women’s decision-making processes regarding participation in a cervical cancer screening program in the Dschang district in West Cameroon.

A qualitative study methodology using focus group discussions was conducted from February to March 2020. Female participants aged between 30 and 49 years and their male partners were invited to participate.

In total, six discussion groups with 43 participants (31 women and 12 men) were conducted. The most important barriers were a lack of health literacy, limited access to the program because of cost and distance, and disrespectful treatment by healthcare workers.

Our results identified three key areas for improvement: first, increasing health literacy; second, providing cervical cancer screening in rural areas; and third, training healthcare providers and community healthcare workers in better patient-provider-communication.

## Introduction

According to the World Health Organization (WHO), 604,127 cervical cancer (CC) cases were diagnosed worldwide, and 341,831 deaths were registered in 2020, most of them occurring in low- and middle-income countries (LMICs) [1]. In sub-Saharan Africa (SSA), including Cameroon, CC is the second leading cause of cancer among women [1, 2]. A total of 2770 new cases were diagnosed in Cameroon in 2020 and 1787 deaths were documented, rendering CC the leading cause of cancer-related deaths among women [2]. Thus, CC is a major public health concern in Cameroon.

Although organized screening programs with high coverage rates have led to a significant reduction in the number of new cases and mortality rates in high income countries, the incidence and mortality rate of CC remains high in Cameroon, and in many LMICs [3, 4]. In response to this situation, the WHO launched a global strategy to accelerate the elimination of CC in November 2020 during the 73rd World Health Assembly. The WHO’s key objectives for 2030 are achieving 90% human papillomavirus (HPV) vaccination coverage for girls, 70% screening coverage and, 90% access to treatment of precancerous and cancerous lesions [5, 6]. Scaling-up or reinforcing these prevention strategies is considered crucial to reduce the gap in health inequalities between high-income and low- and middle-income countries [3].

Aiming to reduce the burden of disease caused by CC in the Dschang district, a 5-year CC screening program was introduced in 2018 at the Dschang District Hospital. However, despite the free provision of clinical services, in the first 6 months the program revealed a 50% lower participation rate than expected [7]. Although previous quantitative studies in SSA have identified a lack of knowledge as an important barrier to CC screening, additional factors may also contribute to the lower participation rate [8–10]. To understand the complex barriers affecting women’s decision processes regarding participation in CC screening, a qualitative study was conducted to explore the perspectives of women and their partners in the Dschang district. The secondary objective of the study was to understand the acceptability and perception of the single visit approach.

## Methods

### Study site

The qualitative data were collected between February and March 2020 in the district of Dschang, located in the west of Cameroon. Dschang city and surrounding areas have an estimated population of approximately 220,000 inhabitants. The study is part of a large trial called the Testing, Triage and Treatment (3T)-Approach, which involves a CC screening program. The 3T-Approach program was implemented in 2018 at the Dschang District Hospital over a 5-year period (2018–2023). This program is a partnership between the University Hospitals of Geneva (Switzerland), University Hospital of Yaoundé (Cameroon), and the University of Dschang (Cameroon) and aims to include 6,000 female participants. The program is supported by the Ministry of Health and is based on a “one day visit” 3T-Approach. The 3T-Approach provides HPV self-sampling, followed by visual assessment for triage of HPV-positive women and treatment by thermal ablation if required, at no cost to participants [4]. HPV self-sampling is one of the three WHO-recommended methods for CC screening. After individual counselling, each woman receives an HPV self-sampling kit and written information, enabling them to collect their own vaginal sample with a dry swab in a private setting. Following rapid HPV testing (Xpert^TM^ HPV), HPV-negative women are reassured and advised to undertake a next screening 5 years later. HPV-positive women undergo visual inspection with acetic acid and visual inspection with iodine (VIA/VILI) and treatment (if indicated) or follow-up [10]. Further details can be found in a recent publication [10].

The study was approved by the Ethical Cantonal Board of Geneva, Switzerland (CCER, N°2017-0110 and CER-amendment n°3) and the Cameroonian National Ethics Committee for Human Health Research (N°2018/07/1083/CE/CNERSH/SP). NCT: 03757299.

### Study setting and design

**Fig. 1:**
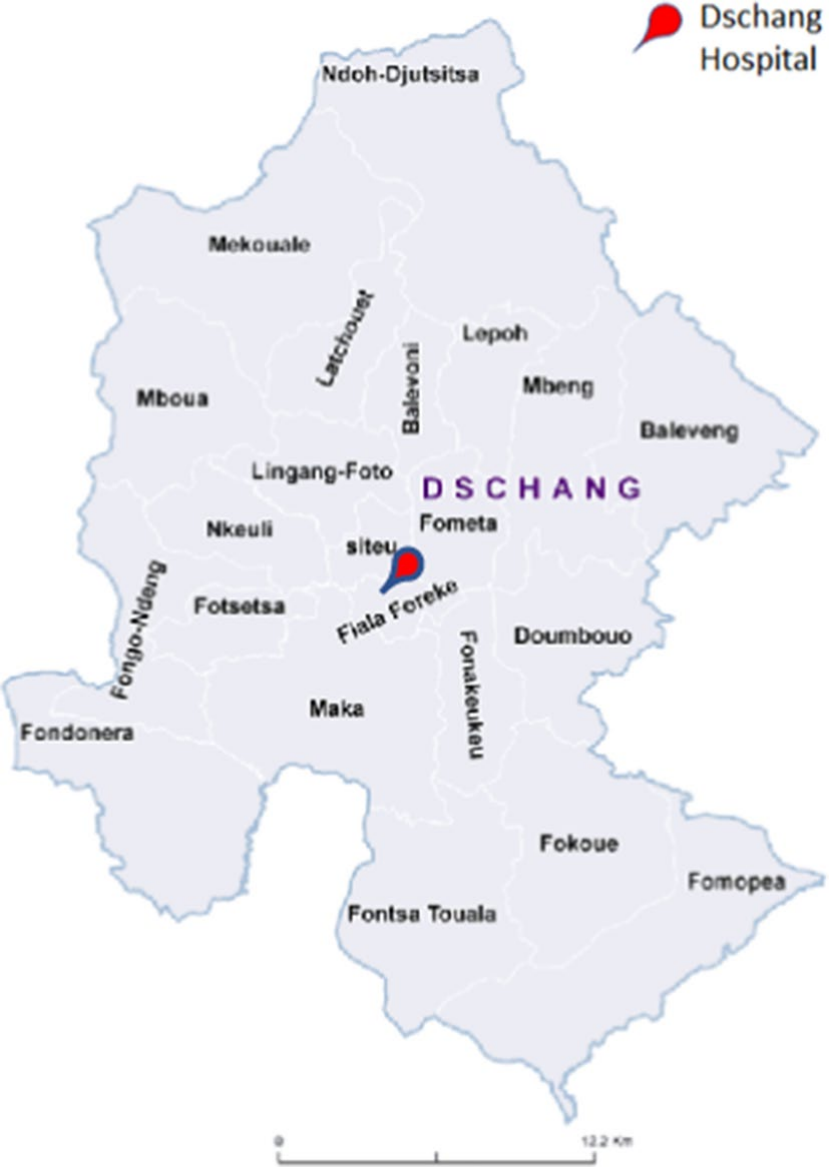
Map of the district of Dschang, West Cameroon, modified from Ministère de la Santé Publique du Cameroun (https://dhis-minsante-cm.org/portal/)

A qualitative methodology was employed, using focus group (FG) discussions with women eligible for the 3T-Approach (inclusion criteria: 30–49 years of age, compliance with the study protocol) and their male partners. FG participants were recruited from three surrounding districts of the Dschang District Hospital, including an urban area (Fiala-Foreke), a semi-urban district (Siteu) and the district of Fometa, which can be considered rural (Fig. 1).

A qualitative methodology using focus groups (FGs) was chosen as an appropriate approach for capturing insights into the ways people perceive and interpret their surroundings [11, 12]. As men often influence women’s decisions about healthcare seeking, positively or negatively, male partners were invited to participate in FGs [13, 14].

Using a semi-structured, pretested interview guide three underlying topics were included to answer the research question: a) knowledge about CC; b) barriers to CC screening; and c) perception and acceptability of the 3T-Approach using HPV-self sampling and providing direct treatment. The FG discussions were conducted either in the home of one of the participants or in a private meeting room provided by the hospitals. As CC is a highly sensitive topic related to sexuality, female health, and gender differences in Cameroon, FGs were conducted in female-only and male-only groups, to allow respondents to communicate freely in group discussions based on shared experiences [12].

Each FG was led by two researchers: one Cameroonian anthropologist (AMD) who facilitated the group discussion in French and one Cameroonian epidemiological student (MEO) who observed group dynamics and body language.

### Recruitment and sampling

We employed non-probability sampling using multiple recruitment strategies. Irrespective of whether they had already attended CC screening, women were invited to participate in the FG by community health workers and personal contacts using a snowball method in three districts. Women living in one of the three districts were eligible to participate in the FGs. At the end of each female FG, women were asked to invite their male partners, male friends, or neighbours to participate in a scheduled FG for men.

In accord with qualitative methodology standards, we applied the principle of theoretical saturation, meaning that no more information related to the main research questions emerged.

### Data analysis

All FGs were recorded after having obtained written consent from each participant. FGs were transcribed and coded using content analysis, using Atlas.ti version 6.1 software. After individual coding, both researchers identified the main and sub-topics common to every group, and outlined the knowledge, perceptions and identified barriers of the women and their partners. Barriers were classified using the conceptual framework of Thaddeus’ and Maine’s three-delay model [15]. This framework was utilized in a previous study exploring the barriers to CC screening in Dschang district from the perspective of healthcare providers, enabling us to compare findings from both studies [16]. According to this model, the decision to seek healthcare can be classified into three delays. The first delay explores factors influencing a woman’s decision-making process, which is affected by her role, the cultural context she is living in, but also the knowledge and the experiences of herself, her family and/or community. The second delay is mainly influenced by factors necessary for reaching the healthcare facility. Such factors include the distance to the facility, road conditions, cost of transportation, and indirect costs, such as being absent from work. The last (third) delay describes factors at the healthcare facility such as the availability of materials or staff. Although the model was originally applied in the context of maternal mortality, it can also be applied to other health situations to identify barriers to screening and assist in the development of appropriate solutions.

## Results

In total, six FGs with 43 participants (31 women and 12 men) were conducted in the three districts; four groups consisted of women only, while two groups consisted of men only.

The FG discussions with a mean of seven participants lasted approximately 40 min. Most participants were married, with an average age of 41 years (range 30–56 years). More than two thirds of participants had completed a minimum of secondary high school education. However, clear gender differences in education were apparent: only four women (13%) reported tertiary education attainment, compared with five of 12 (41%) male participants. Gender differences were also found in occupation: only women worked in the household (11 of 11), women were more likely to work as farmers (16% of women compared to 8% of men) and fewer women worked in professions requiring tertiary education (e.g., as teachers) or were currently studying (12% of women vs. 33% of men) (Table 1: Socio-demographic characteristics).

**Table 1:** Socio-demographic characteristics of FG participants

Variable	N	Percentage (%)
Total	43	100
Gender		
Men	12	28
Women	31	72
Educational level		
Primary school	8	18
Secondary school	26	61
Tertiary education	9	21
Marital status		
Single	4	9
Married/partnership	37	86
Divorced or widowed	2	5
Profession		
Responsible for household	11	25.5
Farmer	6	14
Business	15	35
Other (teacher, student)	11	25.5

### Barriers to CC screening

Barriers to CC screening emerged in all FGs, which were then classified according to the conceptual framework of the three-delay model of Thaddeus and Maine [15]. As described above, although the model was originally suggested to explain factors leading to increased maternal mortality, it can be applied to different health situations because it addresses obstacles influencing individuals’ decisions about seeking healthcare at different levels (i.e., delays). Therefore, the identified barriers can be linked to the individual level (women and their partners) but also the structural level (health-system directly or indirectly) and inform HCPs and policy experts.

### Phase I: delay in decision to seek screening

According to the three-delay model, the healthcare seeking process begins with the decision to seek care. Research has found that various factors will shape women’s decision-making process regarding screening for CC. Among the barriers associated with the decision to seek care, sociocultural factors are most commonly reported in the first delay [15]. The following encountered barriers were reported in our study:

1. Psychological barriers
  Among the female participants, experiences, emotions, and behaviours influenced the decision to seek healthcare. Among these women, the “*fear of the result”* was deemed to be important, as cancer is perceived as a fatal disease in Dschang District.
  “Here at home, cancer is a disease that we are afraid of, because in most cases when we [as a family] have already had family members with blood cancer, breast cancer, prostate cancer, who eventually died. So, it's a disease that scares us.” (Female P11B)
  Participants reported stigma related to fear regarding CC screening results. Women often see themselves as being personally responsible for being diagnosed with CC. A woman from Siteu explained:
  “(…) she went to the hospital. When she comes back, you can see that her appearance has changed. She’s not the way she used to be, because she knows her result was not good. What makes her ashamed now is that she thinks to herself that you know her result, but you don’t.” (Female P7A)
  Furthermore, because previous studies have reported that male partners play an important role in women’s decision-making process, female FG participants were asked about this issue [14]. In contrast to other studies, few women described their spouses as being unsupportive towards them. However, in this situation, relationships were complicated by factors such as substance abuse. A woman from Fiala explained:
  “Influence of the husband: People who are drinkers often do not have time for matters concerning children, or anything that will cost them money.’’ (Female P21C)
2. Knowledge-related barriers
  Both female and male participants believed that a lack of knowledge and/or insufficient information about CC screening was one of the most important barriers. Several female and male participants highlighted that most men and women in the community did not know the causes or symptoms of CC. Emphasis was placed on the lack of understanding, particularly regarding knowledge about prevention strategies and treatments for CC at an early stage. A man from Fiala explained:
  “…it is a disease that seems new to us. When we were young, we didn’t hear about it, but today we are told that there is cancer that attacks the cervix. It bothers us that we do not know where it comes from.” (Male P25B)

### Phase II: delay reaching the screening centre

Two important barriers emerged from the FGs with both women and men: the financial cost of attending the CC screening program and the time required to reach the healthcare facility where the CC screening program was offered.

Respondents of both genders mentioned direct and indirect costs as important barriers to attending screening. Even if women were aware that the CC screening program was free of charge and perceived this as an important motivational factor, the additional costs of transportation, being absent from work, and having to take care of their children, were still an issue.

The district hospital of Dschang is one of the few facilities that offers CC screening in Western Cameroon. Hence, women, especially those who live in rural areas, face a double burden in respect to healthcare: cost and the difficulty of reaching the facility.

### Phase III: receiving adequate and appropriate screening and treatment

The third delay involves factors related to the quality of healthcare at the facility, which can be divided into technical quality and patient experiences. Insufficient technical quality refers to shortages of supplies but also the direct application of clinical services. On the other hand, patients’ experiences include non-health needs. In a working paper for the World Health Organization, Gostin et al. defined eight domains of health responsiveness, which included (1) respect for the dignity of persons; (2) autonomy to participate in health-related decisions; (3) confidentiality; (4) prompt attention; (5) adequate quality of care; (6) communication; (7) access to social support networks; and (8) choice of healthcare providers [17].

In our study, FG participants identified in this phase inadequate health communication and disrespectful treatment by HCPs as the two most important barriers to the CC screening program. Participants highlighted that information regarding CC screening needed to be communicated effectively and in a way that could be easily understood by both women and men. Participants in our study also emphasised that healthcare providers (HCPs) need to acknowledge that, besides time and money, the decision to attend screening requires courage, due to the fear of a positive result after screening (see delay 1). Therefore, all women attending screening should be acknowledged by HCPs and treated with respect on the day of their consultation.

In addition, a woman’s prior or current experience with an HCP that did not treat her with respect may render her less likely to access the CC screening program and reduce the chances of her returning for a follow-up visit.

“You know others initially traumatize people. For example, the woman [referring to a female HCP] who was recording there, she [….] asks Poupoupou questions (brutally/quickly)! [laughs] [….] She stresses you out by asking the questions quickly. No, that’s not the way to do it. She needed to slow down a bit.” (Female P13B)

Furthermore, additional negative experiences with HCPs or insufficient information (for example, information regarding the number of days or length of the CC screening) were reported, potentially causing women to actively discourage other women to get screened.

The secondary objective of the study was to understand the acceptability and perception of the single visit approach (3T). While none of the female participants referred to the fact that screening and treatment were offered during the same visit, several women expressed mixed attitudes towards the HPV self-sampling method. While the intention of the HPV self-sampling is to reduce shame and provide more intimacy by enabling women to collect their own vaginal HPV swab, some women perceived it as a way for the program to work more effectively and save HCP’s time. Others questioned their ability “to do it right” and would have appreciated clear support from HCPs, as a woman from Fiala explained:

“I would still have suggested that they should form a team to help those *who do not know how to do it.”* (Female P41C)

However, while men understood their partners’ concerns, they also perceived the HPV self-sampling method as an adequate way to protect their “wife’s nudity”. A man from Fometa said:

“I choose the method where it is the woman herself who takes it. [Laughs] When she samples it herself, she's not even ashamed since she’s doing it alone. But there are women who are even ashamed to examine their sexual parts in private.” (Male P26B)

The results of the FG discussion revealed that barriers to attend CC screening existed in all three delays. The following section will discuss the encountered barriers in the context of the current literature and suggest possible interventions to overcome them.

## Discussion

To the best of our knowledge, the current study is one of the few qualitative studies conducted in Cameroon that aims to understand the barriers to attending a CC screening program using the 3T approach, and to suggest possible solutions. Most of the following discussion will focus on encountered barriers at the micro level (HCPs or patients) or meso level (healthcare institutions) that can be addressed by the CC program itself. In this sense, the three most important barriers encountered in all three delays were: (1) knowledge-related barriers (2) difficulties reaching the healthcare centre and (3) disrespectful treatment by healthcare staff. Therefore, the following improvements to the CC program should be made: (1) the advancement of health literacy (at the user and the provider side), (2) the delivery of CC screening and (3) the provision of respectful healthcare.

In the following discussion, we will discuss these principal findings in relation to the findings of previous studies.

- *Enhancing health literacy*
  The identified lack of awareness or insufficient knowledge is associated with a lack of health literacy. Health literacy has been defined by the WHO as ‘‘the cognitive and social skills which determine the motivation and ability of individuals to gain access to, understand and use information in ways which promote and maintain good health” [18]. As shown in the current study, several participants were not aware of organizational aspects of the CC program (such as days of screening or duration of consultation) which inhibited their ability to “use information” and “maintain good health”.
  Lack of health literacy was noted to be greatest in rural areas in which education was lower and additional barriers due to the financial constraints held greater weight. Kim et al. reported that increasing health literacy is the first step in promoting CC screening programs [19]. In this review Kim and colleagues explore the linkage between CC screening behaviours and health literacy. The review indicated that lower participation rates were linked with low knowledge about CC, as well as social determinants beyond health, such as education. Therefore, increasing health literacy in the Cameroonian context entails not only the provision of knowledge about CC symptoms, but also education about CC prevention. Women in our study reported difficulty attending screening for a disease that they did not know about. In addition, respondents were not aware of the importance of prevention and detection of CC at an early stage. This observation is in line with previous studies conducted in Africa, Europe, and Asia, highlighting the need to carry out community education for women and men about the importance of preventive screening, because precancerous lesions are often asymptomatic [20–27]. In addition, in a study conducted by Roux et al. to examine the CC screening program in Dschang, HCPs reported that women’s misconceptions about CC symptoms and prevention strategies explained why women did not access CC screening. According to Roux et al., improving health literacy also encompasses addressing fatalistic perceptions and stigma [16]. Because CC is perceived to be fatal, and sometimes viewed as a punishment, screening can stimulate emotions such as fear and shame. Consequently, it is important to discuss stigma as a barrier to CC screening and prevention [23, 25, 26].
- *Improving delivery of CC screening*
  Nearly all female and male participants mentioned that difficult living conditions act as a barrier, particularly poverty and distance to CC screening facilities. The role of distance is a major barrier in the decision to seek treatment. Previous studies have reported that the disparity between rural and local areas is exacerbated by poverty. [4, 16, 25] Participants identified mobile screening facilities as a practical way to improve physical and economic access to CC screening. A female participant explained:
  “If we come to find you there, we sacrifice ourselves; we close our shops, we know that we are sacrificing ourselves (…). When we were tested for HIV, no one left. They came here, they tested over a hundred people. Whereas if we said we were going to the hospital nobody was going to leave. …” (Female P6A).
  However, even if mobile screening options could address the factors mentioned in the second delay (cost and distance), comprehensive community strategies remain critical for improving women and men’s health literacy and supporting women’s decision-making processes regarding attending CC screening. Furthermore, participants highlighted the importance of CC awareness campaigns using personal contact, but also mass media, such as radio and television, which previous studies have reported to be used successfully [28, 29].
- *Provision of respectful healthcare*
  Healthcare utilization has been linked to the quality of care patients receive. The current study revealed barriers to CC screening, particularly in respect to communication and respect towards patients. Female participants not only outlined the previously described organizational aspects of the CC screening program, they also reported that disrespectful communication of HCPs negatively influenced their decision to access CC screening. As Larson et al. reported in a recent study in seven African countries, poor provider communication is linked to lower satisfaction, influencing patients’ likelihood of returning for a follow-up exam [8]. Improved communication skills could also address concerns about the application of the HPV-self-test, which was highlighted by several women. This possibility is in accord with previous studies reporting that women’s concerns about the self-HPV test were present irrespective of their economic situation [30, 31]. Interestingly, a recent study reported a positive correlation between a patient’s experience and the level of education of HCPs, as well as the patient’s educational level [8]. We propose two possible explanations for this association. First, patients with a higher level of education are more likely to understand what is being said. Second, HCPs may use language based on the educational level of the users, hence increasing the patient’s understanding of the consultation with the HCP. Because of the qualitative nature of our study, we were unable to test for this correlation. However, as the education level in the Dschang District/Cameroon is relatively low, it would be useful for future research to explore this association. This applies also to the involvement of community healthcare workers in the CC screening program. Community healthcare workers play an important role in motivating women to attend CC screening. However, as their educational level is often relatively low, training should include communication skills, with a focus on the importance of always treating patients with respect.
  The current qualitative study is among the first to explore barriers to CC screening in the rural area of Dschang. However, several limitations should be acknowledged. First, due to its qualitative methodology, the range of topics considered important by the FG participants does not necessarily reflect their relative importance in the population. Second, the FG methodology might have influenced some participants to give answers that they thought were socially acceptable. Nonetheless, because all FGs were conducted by an experienced anthropologist from Cameroon in a confidential location, we believe that most participants felt comfortable expressing their personal opinions. Finally, although extensive efforts were made to include women and men from different settings (rural vs. urban, with diverse socio-economic characteristics), the sample size was relatively small, and the possibility of selection bias cannot be excluded.

## Conclusion

Despite the limitations of our study, the current results corroborate many previous studies and are therefore considered to be generalizable for similar settings. The framework of the three-delay-model of Thaddeus and Maine allowed us to identify barriers to CC screening at the micro- and meso-levels in the Dschang district in all three delays. While barriers in the first two delays, including knowledge- and distance-related barriers, have been reported in previous studies in SSA, our study highlighted the importance of improving the quality of healthcare provided, especially in respect to communication. Reducing identified barriers may be beneficial at the personal and institutional levels, supporting health system strategies to improve health equity.

Therefore, the following key strategies are suggested: (1) enhancing health literacy by strengthening community health activities; (2) improving the delivery of CC screening activities in rural areas; and (3) providing training for HCPs and community healthcare workers to improve patient-provider-communication.

## Data Availability

The datasets (transcripts) generated and analysed during the current study are not publicly available due to the sensitivity of the data. The interview guide or summaries of transcripts, including categories and codes, can be made available from the corresponding author upon reasonable request.

## Abbreviations

FG: Focus group
CC: Cervical cancer
HCP: Healthcare provider
HIV: Human immunodeficiency virus
HPV: Human papillomavirus
LMICs: Low- and middle-income countries
SSA: Sub-Saharan Africa
VIA/VILI: Visual inspection with acetic acid and visual inspection with iodine
WHO: World Health Organization
